# Profil des cas de mpox identifiés lors de la surveillance de la zone de santé Kokolo à Kinshasa (RDC) d'août à novembre 2024

**DOI:** 10.48327/mtsi.v5i1.2025.604

**Published:** 2025-03-25

**Authors:** Levis AMISI KENGEA, Blanche IHEKAMBANGU NGWAKAHA, Winnie MASAMBA BIKOKI, Vally NDUMBI TEMUANGUDI, Jean Claude NSINGA BUNGIENA, Jean-Jacques KAPE KALUME, Anthony MBUYI MUTOMBE, Angèle WUMBA MAVINGA

**Affiliations:** 1 Zone de santé Kokolo, Corps de santé militaire, Forces armées de la République démocratique du Congo; 2 Hôpital militaire central, Corps de santé militaire, Forces armées de la République démocratique du Congo; 3 Centre médical opérationnel de type A de la 113ème base aérienne de Ndolo, Corps de santé militaire, Forces armées de la République démocratique du Congo, République démocratique du Congo; 4 Corps de santé militaire, Forces armées de la République démocratique du Congo

**Keywords:** Mpox, Surveillance, Kokolo, Kinshasa, République démocratique du Congo, Afrique subsaharienne, Mpox, Surveillance, Kokolo, Kinshasa, Democratic Republic of the Congo, Sub-Saharan Africa

## Abstract

**Introduction:**

La zone de santé militaire Kokolo dans la ville de Kinshasa (RDC) fait partie de celles touchées par le mpox, avec la présence de deux sous-clades Ia et Ib du virus de la variole du singe. Cette étude présente les résultats des activités de surveillance liées au mpox dans cette zone.

**Méthodes:**

Étude descriptive des données rapportées par le système de surveillance sur l’épidémie de mpox dans la zone de santé Kokolo entre août et novembre 2024.

**Résultats:**

Sur 202 échantillons (143 hommes, sex-ratio 0,71) chez des individus cliniquement suspects de mpox ou contacts de cas connus, 25,2 % étaient positifs en PCR au laboratoire de l’Institut national de recherches biomédicales (âge médian 25 ans, IIQ 11-31). Les sujets de 18 ans et plus étaient les plus touchés soit 41/51 (80 %). Sur ces 51 cas confirmés, 13 (26 %) et 10 (20 %) provenaient respectivement des aires de santé Kokolo 2 et base logistique dans le camp Kokolo. Tous les cas confirmés présentaient des éruptions cutanées. Les autres symptômes signalés étaient des myalgies (50/51, 98 %), des éruptions génitales (42/51, 82 %), des arthralgies (42/51, 82 %) et des céphalées (41/51, 80 %). La durée médiane de séjour dans le centre de traitement du mpox a été de 10 jours (IIQ : 7-9).

**Conclusion:**

Cette étude a montré que sur 202 tests effectués sur des personnes suspectes de mpox ou contacts de cas connus, 25,2 % étaient positifs au test PCR. Tous les cas confirmés présentaient des éruptions cutanées, avec d'autres symptômes fréquents comme myalgies, éruptions génitales, arthralgies et céphalées. Ces résultats mettent en évidence la nécessité de renforcer les mesures de surveillance et de contrôle de la propagation de la mpox, en particulier dans les aires de santé les plus touchées.

## Abstract (en)

**Introduction:**

The Kokolo military health zone in the city of Kinshasa (DRC) is one of the areas affected by mpox, with the presence of two subclades Ia and Ib of the monkeypox virus. This study presents the results of mpox-related surveillance activities in this area.

**Methods:**

Descriptive study of data reported by the surveillance system on the mpox epidemic in the Kokolo health zone between August and November 2024.

**Results:**

Of 202 specimens (143 males, sex ratio 0.71) from individuals clinically suspected of having mpox or contacts of known cases, 25.2% were positive by PCR at the National Institute for Biomedical Research laboratory (median age 25 years, IQR 11-31). Subjects aged 18 years and older were most affected, i.e. 41/51 (80%). Of these 51 confirmed cases, 13 (26%) and 10 (20%) were from the Kokolo 2 health areas and the Kokolo camp logistics base, respectively. All confirmed cases presented with rash. Other reported symptoms were myalgia (50/51, 98%), genital rash (42/51, 82%), arthralgia (42/51, 82%), and headache (41/51, 80%). The median length of stay at the mpox treatment center was 10 days (IQR: 7-9).

**Conclusions:**

This study showed that of 202 tests performed on persons suspected of having mpox or contacts of known cases, 25.2% were positive by PCR. All confirmed cases presented with rash, with other common symptoms including myalgia, genital rash, arthralgia, and headache. These results underscore the need to strengthen surveillance and control measures for the spread of mpox, particularly in the most affected health care settings.

## Introduction

Le mpox, ou variole simienne, est une infection zoonotique décrite pour la première fois en République démocratique du Congo (RDC) en 1970 [1, 4]. Historiquement, le virus de la variole du singe (MPXV) était présent dans les forêts tropicales d’Afrique centrale et occidentale, où il est reconnu comme endémique [7, 14]. On le divise en deux clades : le clade I, précédemment appelé clade du bassin du Congo (Afrique centrale) et le clade II, précédemment désigné comme clade ouest-africain. Les sous-clades Ia et Ib sont des variantes du virus du mpox. Le sous-clade Ia est principalement associé à des transmissions à partir d'animaux sauvages. Les chaînes de transmission interhumaine de ce sous-clade sont généralement limitées et se produisent principalement dans les zones rurales et forestières endémiques alors que le sous-clade Ib se caractérise par une transmission interhumaine soutenue, surtout dans les zones urbaines. Il a été détecté dans plusieurs pays, y compris en dehors de l’Afrique, et a été responsable de plusieurs flambées épidémiques. Ce sous-clade est une préoccupation pour la santé publique en raison de sa capacité à se propager rapidement parmi les populations humaines. Le clade II se divise également en deux sous-clades : IIa et IIb. Le sous-clade IIa est principalement trouvé en Afrique centrale et occidentale. Il a été associé à des flambées épidémiques locales mais pas à une pandémie en 2022-2023 alors que le sous-clade IIb est responsable d'une flambée mondiale de mpox. Celui-ci a été détecté dans de nombreux pays à travers le monde et a montré une capacité accrue de transmission interhumaine, en particulier dans les zones urbaines. Ces sous-clades soulignent l'importance de la surveillance continue et des mesures de contrôle pour prévenir la propagation de la maladie [10, 12, 13].

La zoonose a joué un rôle prédominant dans les infections par le MPXV chez l'humain, provoquant également des infections secondaires liées au contact entre individus. Toutefois, lors de la pandémie de 2022, les cas d'infection par le MPXV du sous-clade IIb se sont principalement manifestés par une transmission interhumaine continue [9], notamment dans la communauté des hommes qui ont des relations sexuelles avec des hommes [4, 11]. L'apparition du sous-clade Ib au Sud-Kivu, en RDC, a révélé des caractéristiques de transmission similaires, incluant une corrélation avec les réseaux sexuels et une transmission intense entre les individus [4, 14].

La récente expansion du sous-clade Ib impliquant des enfants dans le Nord-Kivu a suggéré une possible transmission par des relations non sexuelles. Des biais de signalement peuvent entraver une compréhension plus approfondie de l’épidémiologie de la variole simienne. Les mesures de confinement et d'atténuation de la transmission dans ce contexte d'urgence sanitaire doivent considérer les particularités propres au clade de MPXV [4]. L’épidémie continue de se propager dans d'autres pays africains, notamment l’Afrique du Sud, la Côte d’Ivoire, le Kenya, le Liberia, le Nigeria, l’Ouganda, la République centrafricaine, le Burundi et le Rwanda. Cela a conduit au déclenchement de la première alerte sanitaire pour la sécurité du continent par le Centre de contrôle africain, et au déclenchement de la seconde alerte sanitaire internationale pour la variole simienne par l’Organisation mondiale de la Santé (OMS). En RDC, le mpox est désormais considéré comme une épidémie majeure au niveau national, affectant également la province de Kinshasa. Dix-sept de ses 35 districts sanitaires ont déjà subi des dommages, y compris la zone de santé militaire Kokolo. C'est une zone spécifique qui dispose d'espaces de santé (camps militaires) répartis dans toute la ville-province de Kinshasa. Elle prend en charge les militaires, les dépendants des militaires et la population environnante des camps militaires. L'expansion du sous-clade Ib est contemporaine d'une augmentation des cas dans cette zone de santé. Nous proposons une étude des cas de mpox dans cette zone de santé basée sur les informations de surveillance entre août et novembre 2024.

## Méthodes

La résolution AFR/RC48/R2 adoptée par le Comité régional de l’OMS pour l’Afrique lors de sa 48^e^ session sert de référence administrative pour l'organisation de la surveillance épidémiologique en RDC. Cette résolution demande aux États-membres de procéder à une évaluation de la composante laboratoire des programmes de lutte contre les maladies, comme première étape vers le renforcement de la surveillance. Elle met l'accent sur l'importance des laboratoires de santé publique pour fournir des informations fiables et en temps opportun pour la prise en charge des patients et la surveillance des maladies. Elle vise à améliorer les capacités des laboratoires afin de mieux détecter et répondre aux épidémies de maladies telles que la fièvre à virus Ébola, la fièvre de Marburg, la tuberculose et les maladies éruptives comme le mpox [8].

Dans la zone de santé Kokolo, plusieurs types de surveillance sont organisés : surveillance passive, surveillance basée sur la population et surveillance à base communautaire. La recherche active des cas de mpox s'y est ajoutée. La surveillance est réalisée de la base au sommet simultanément dans les établissements des soins de santé (ESS) et dans les communautés (églises, écoles, lieux de travail, etc.). Chaque lundi à 12 heures au plus tard, les ESS doivent rapporter au Bureau central de la zone de santé (BCZS) les données hebdomadaires de surveillance à l'aide d'une tablette et d'une fiche de rapport hebdomadaire. Cependant, la notification de tout cas suspect de mpox est instantanée, à l'aide d'un appel téléphonique ou d'un message sms ou WhatsApp. Le BCZS Kokolo compile toutes les données venues des aires de santé pour les envoyer à la Division provinciale de la santé de Kinshasa au plus tard le mardi à 12 heures. L'alerte communautaire ou dans l’ESS passe par l'infirmier titulaire qui investigue à son niveau puis fait remonter l'alerte au BCZS. Celui-ci valide ou non l'alerte. Si l'alerte est invalidée, le sujet est pris en charge pour autre chose que le mpox. Si l'alerte est validée, le cas suspect est isolé dans un centre de traitement du mpox (CTmpox) et bénéficie d'un prélèvement sanguin, de croûte ou nasopharyngé, selon le stade de la maladie. Cet échantillon est acheminé au laboratoire de l’Institut national des recherches biomédicales (INRB) pour une analyse par PCR. Le sujet suspect quitte le CTmpox après guérison ou si le résultat de laboratoire revient négatif. Les analyses sont toujours faites au niveau du laboratoire de l’INRB sur la base des échantillons prélevés par les techniciens du laboratoire. Les résultats arrivent dans les trois jours au maximum. Ce court délai permet la réalisation des actions de santé publique : élargissement des listes de contacts, suivi des contacts pendant 21 jours à partir du jour de dernier contact avec le cas confirmé, décontamination des endroits parcourus par le malade pendant sa phase symptomatique, sensibilisation des contacts et personnes proches du cas confirmé, soutien psychosocial des contacts et personnes proches du cas confirmé, vaccination de tous les contacts [2].

La présente analyse est une étude descriptive des données rapportées sur l’épidémie de mpox dans la zone de santé Kokolo du 1^er^ août 2024 au 10 novembre 2024. Elles sont extraites des bases de riposte à la variole simienne du district.

- Cas alerte : toute personne ayant une forte fièvre d'apparition soudaine et qui ne s'arrête pas après le traitement initial, ou toute personne ayant eu des éruptions cutanées ou muqueuses.
- Cas suspect : toute personne qui a été en contact avec un cas de mpox et qui a une fièvre aiguë (≥38 °C) ou qui présente une fièvre aiguë et des symptômes de mpox, ou toute personne qui a une ou plusieurs éruptions maculo-papulaires ou des croûtes.
- Cas probable : un cas suspect (décédé ou non retrouvé) pour lequel un résultat de laboratoire pour le mpox n'a pas pu être obtenu mais ayant généré des cas contacts. Il s'agit d'un patient qui, pour des raisons cliniques et/ou épidémiologiques, présente une très forte probabilité d’être infecté par le virus de mpox mais que le laboratoire n'a pas confirmé.
- Cas confirmé : tout cas suspect pour lequel un test de laboratoire (PCR) est positif.
- Non cas : tout cas pour lequel le test de laboratoire est négatif [2, 6].

L’échantillon étudié est constitué des cas enregistrés dans la base de données de riposte de la zone de santé Kokolo. La collecte des données a été faite à partir de la liste des cas de mpox du district sanitaire de Kokolo et des feuilles de rapport des maladies à potentiel épidémique.

Cette étude a été exemptée d'approbation éthique puisqu'elle a été menée dans le cadre des activités de surveillance nationale et réalisée dans l'intérêt public par le ministère de la Santé de la RDC. Toutes les activités décrites ont été entreprises dans le cadre d'une surveillance régulière menée et approuvée directement par le ministère de la Santé. Aucun consentement éclairé écrit n'a été demandé, car les analyses effectuées dans le cadre de cette étude ont été effectuées rétrospectivement à partir des informations et des échantillons recueillis à des fins de surveillance et de soins.

## Résultats

Au cours de la période d’étude, 47 prélèvements et tests ont été effectués en août, 58 en septembre, 62 en octobre et 35 en novembre 2024, soit un total de 202 tests effectués par PCR pour recherche du MPXV. Dans les 202 tests, 123 concernaient des militaires dont 41 confirmés mpox (33 %), 65 des dépendants des militaires, dont 9 confirmés mpox (14 %), et 14 la population environnante des camps militaires, dont 1 confirmé mpox.

Tous les cas confirmés de mpox présentaient des éruptions cutanées. Les autres symptômes signalés sont des myalgies (50/51), des éruptions génitales (42/51), des arthralgies (42/51) et des céphalées (41/51) (Tableaux I, 2, 3 and IV). Deux patients parmi les hommes admis au CTmpox présentaient une séropositivité au VIH, 2 cas d'hypertension artérielle étaient relevés, 1 cas d'asthme et 1 cas de diabète sucré.

**Tableau I:** Répartition des résultats de test de mpox par sexe dans la zone de santé Kokolo, d'août à novembre 2024

	Positif	Négatif	Indéterminé
**Sexe masculin**	36	104	3
**Sexe féminin**	15	42	2
**Total**	51 (25,2 %)	146 (72,3 %)	5 (2,5 %)

**Tableau II:** Répartition des personnes testées pour le mpox par âge dans la zone de santé Kokolo, d'août à novembre 2024

	Âge médian (ans) Résultat mpox positif	Âge médian (ans) Résultat mpox négatif
**Sexe masculin**	29 (IIQ : 11-31)	28 (IIQ : 17-44)
**Sexe féminin**	20 (IIQ : 14-30)	19 (IIQ : 11-30)
**Total**	25 (IIQ : 11-31)	24 (IIQ : 16-44)

**Tableau III:** Répartition des personnes atteintes de mpox confirmé par âge et par sexe dans la zone de santé Kokolo, d'août à novembre 2024

	Sexe masculin n (%)	Sexe féminin n (%)	Total n (%)
**< 15 ans**	4 (8 %)	2(4 %)	6 (12 %)
**15-30 ans**	15 (29 %)	13 (25 %)	28 (54 %)
**≥30 ans**	16 (31 %)	1 (2 %)	17 (34 %)

**Tableau IV:** Hospitalisations, données démographiques et symptômes cliniques identifiés dans la zone de santé Kokolo, d'août à novembre 2024

Groupe d’âge (ans) n = 202	Sexe masculin n (%)	Sexe féminin n (%)	Total n (%)
**< 15 ans**	17 (8,4 %)	7(3,5 %)	24 (11,9 %)
**15-30 ans**	61 (30,2 %)	49 (24,3 %)	110 (4,4 %)
**≥30 ans**	65 (32,2 %)	3 (1,5 %)	68 (33,7 %)
**Total**	143 (70,8 %)	59 (29,2 %)	202 (100 %)
**Âge médian (ans)**	25 (IIQ : 4-38)	24 (IIQ : 5-35)	24 (IIQ : 4-38)
**Durée des soins (jours)**	9 (IIQ : 6-12)	11 (IIQ : 8-13)	10 (IIQ : 6-11)
**Hospitalisation**	32/35	14/16	46/51 (90 %)
**Symptômes cliniques n=51**			
**Éruptions cutanées**	35	16	51 (100 %)
**Éruptions génitales**	29	13	42 (82 %)
**Éruptions buccales**	16	15	31 (61 %)
**Fièvre**	24	12	36 (71 %)
**Céphalées**	29	12	41 (80 %)
**Myalgies**	35	15	50 (98 %)
**Arthralgies**	29	13	42 (82 %)

## Discussion

Depuis janvier 2023, le développement considérable du MPXV du clade I en RDC a provoqué une importante épidémie de mpox dans toutes les provinces du pays. Un sous-clade inédit du virus, le sous-clade Ib, lié à une transmission interhumaine est apparu [14]. Son premier signalement a eu lieu dans la province du Sud-Kivu. Il s'est rapidement propagé au Nord-Kivu [5, 7], ainsi que dans la province de Kinshasa, principalement dans la zone de santé Kokolo où il s'agit de la première épidémie de mpox. En 2023, cette zone de santé n'avait enregistré que deux cas de mpox dont un décès. Le seul type de transmission noté était interhumaine avec le sous-clade Ia. Mais lors de l’épisode d'août-novembre 2024, 51 cas confirmés par PCR ont été enregistrés appartenant aux deux sous-clades Ia et Ib, présentant des éruptions aux niveaux des voies génitales externes.

Les systèmes de surveillance dans toutes les zones de santé de RDC sont théoriquement les mêmes, mais les analyses des données de surveillance tout comme de riposte ne sont pas toujours régulièrement tenues à jour. Auparavant, le mpox était considéré comme une maladie atteignant surtout les enfants [3, 6, 13]. Dans notre étude, les adultes sont davantage concernés. Les militaires apparaissent plus touchés, probablement en lien avec leur profession qui les expose aux personnes de tout genre et tout lieu, dont les professionnels du sexe.

La durée d'hospitalisation a probablement été raccourcie en raison du nombre insuffisant de CTmpox dans la ville de Kinshasa (3 seulement pour une population de plus de 10 millions d'habitants).

Les atouts du système de surveillance sont :

- le système existe et il est fonctionnel, de la communauté au secrétariat général voire au cabinet du ministre de la Santé avec une rétro-information à chaque niveau;
- il comprend des acteurs formés pour plusieurs maladies;
- les structures de surveillance sont en place avec un circuit tracé défini.

Cependant, ce système connaît des limites :

- il nécessite une mise à jour continue des connaissances et des compétences, des définitions de cas selon le contexte et une bonne coordination pour plus d'efficacité;
- le système est voué à l’échec sans l'adhésion de la communauté;
- le système nécessite des moyens appropriés en permanence au niveau des bureaux centraux des zones de santé et établissements de soin de santé.

Nous formulons des recommandations à différents niveaux :

Division provinciale de la Santé :

- améliorer les conditions de travail des acteurs à tous les niveaux;
- actualiser les définitions des cas;
- améliorer la coordination de riposte à tous les niveaux.

Partenaires techniques et financiers de la zone de santé :

- doter la zone de santé Kokolo d'au moins troisappareils Genexpert pour couvrir les trois axes de celle-ci;
- développer la capacitation continue et permanente des acteurs sur tous les plans et à tous les niveaux;
- rendre disponibles les moyens de lutte;
- améliorer les conditions de travail des acteurs à tous les niveaux;
- améliorer la coordination de riposte à tous les niveaux.

3. Aire de santé :

- améliorer les conditions de travail des acteurs;
- améliorer la sensibilisation et la communication pour obtenir l'engagement communautaire.

4. Communauté : faire adhérer complètement la communauté dans les activités de riposte au mpox. Ces recommandations sont cruciales pour renforcer la lutte contre le mpox et améliorer la réponse sanitaire au niveau du district sanitaire. Cela nécessite une collaboration étroite entre tous les niveaux pour assurer une mise en œuvre efficace et durable.

## Conclusion

Cette étude a montré que sur 202 tests faits sur des personnes cliniquement suspectes de mpox ou contacts de cas connus, 25,2 % étaient positifs au test PCR. Les adultes de 18 ans et plus étaient les plus touchés, représentant 80 % des cas confirmés. Tous les cas confirmés présentaient des éruptions cutanées, avec d'autres symptômes fréquents comme myalgies, éruptions génitales, arthralgies, céphalées. La durée médiane de séjour dans le centre de traitement du mpox était de 10 jours. La détection tardive des cas montre qu'il y a soit des difficultés dans le suivi des contacts, soit que la définition des cas devrait être revue pour permettre leur détection précoce dans la communauté et parmi les contacts. Il faut également s'assurer que l’élargissement de la liste des contacts et le suivi de ces derniers pendant 21 jours soient effectifs. Les sujets de sexe masculin, les sujets jeunes souvent militaires étant les plus touchés, les actions doivent être orientées vers ces catégories.

## Source de financement

Les auteurs ne déclarent aucune source de financement.

## Contributions des auteurs et autrices

IHEKAMBANGU NGWAKAHA Blanche : analyses et rédaction.

MASAMBA BIKOKI Winnie : conception, analyses et rédaction.

NDUMBI TEMUANGUDI Vally : conception, analyses et rédaction.

NSINGA BUNGIENA Jean Claude : analyses et rédaction.

KAPE KALUME Jean-Jacques : rédaction. MBUYI MUTOMBE Anthony : analyses et rédaction.

WUMBA MAVINGA Angèle : rédaction.

AMISI KENGEA Levis : auteur correspondant, conception, revue de la littérature, disponibilité des données, analyses, rédaction du rapport et de l'article.

## Conflit d'intérêt

Les auteurs déclarent ne pas avoir de liens d'intérêts.

WUMBA MAVINGA Angèle: writing.

AMISI KENGEA Levis: corresponding author, design, literature review, data availability, analyses, report and writing.
