# Supplementary material for: Profile of mpox cases identified during surveillance of the Kokolo health zone in Kinshasa (DRC) from August to November 2024
Source: Med Trop Sante Int. 2025 Mar 25;5(1):mtsi.v5i1.2025.604. [Article in French] doi: 10.48327/mtsi.v5i1.2025.604 (PMC12001985; doi:10.48327/mtsi.v5i1.2025.604)
Supplement: Related texts

# Research article: Profile of mpox cases identified during surveillance of the Kokolo health zone in Kinshasa (DRC) from August to November 2024

## Introduction

Mpox, or monkeypox, is a zoonotic infection first described in the Democratic Republic of the Congo (DRC) in 1970 [1, 4]. Historically, monkeypox virus (MPXV) was present in the tropical forests of Central and West Africa, where it is recognized as endemic [7, 14]. It is divided into two clades: clade I, formerly known as the Congo Basin (Central Africa) clade, and clade II, formerly known as the West African clade. Subclades Ia and Ib are variants of the mpox virus. Subclade Ia is primarily associated with wildlife transmission. Human-to-human transmission chains of this subclade are generally limited and occur mainly in rural and forested endemic areas, while subclade Ib is characterized by sustained human-to-human transmission, particularly in urban areas. It has been detected in several countries, including outside Africa, and has been responsible for several outbreaks. This subclade is of public health concern because of its ability to spread rapidly among human populations. Clade II is also divided into two subclades: IIa and IIb. Subclade IIa is found primarily in Central and West Africa. It has been associated with local outbreaks but not with a pandemic in 2022-2023, while subclade IIb is responsible for a global outbreak of mpox. The latter has been detected in many countries around the world and has shown an increased capacity for human-to-human transmission, particularly in urban areas. These subclades highlight the importance of continued surveillance and control measures to prevent the spread of the disease [10, 12, 13].

Zoonotic transmission has played a predominant role in human MPXV infections, including secondary infections associated with person-to-person contact. However, during the 2022 pandemic, cases of MPXV subclade IIb infection mainly manifested as continuous human-to-human transmission [9], particularly in the community of men who have sex with men [4, 11]. The emergence of subclade Ib in South Kivu, DRC, has revealed similar transmission characteristics, including a correlation with sexual networks and intense inter-individual transmission [4, 14].

The recent expansion of subclade Ib to include children in North Kivu suggests possible transmission through non-sexual relationships. Reporting biases may prevent a more thorough understanding of the epidemiology of monkeypox. Measures to contain and reduce transmission in the context of this public health emergency must take into account the specificities of the MPXV clade [4].

The epidemic continues to spread to other African countries, including South Africa, Côte d’Ivoire, Kenya, Liberia, Nigeria, Uganda, Central African Republic, Burundi and Rwanda. This led to the first health alert for the safety of the continent by the Africa Monitoring Center and the second international health alert for monkeypox by the World Health Organization (WHO).

In the DRC, mpox is now considered a major epidemic at the national level, including the province of Kinshasa. Seventeen of its 35 health districts have already been affected, including the Kokolo military health zone. This is a specific zone with health facilities (military camps) spread throughout the city-province of Kinshasa. It serves the military, the military's dependents and the population surrounding the military camps. The spread of subclade Ib coincides with an increase in cases in this health zone. We propose a study of mpox cases in this health zone based on surveillance information between August and November 2024.

## Methods

Resolution AFR/RC48/R2, adopted by the WHO Regional Committee for Africa at its 48^th^ session, serves as an administrative reference for the organization of epidemiological surveillance in the Democratic Republic of the Congo. The resolution calls on Member States to carry out an assessment of the laboratory component of disease control programmes as a first step towards strengthening surveillance. It emphasizes the importance of public health laboratories in providing reliable and timely information for patient mangement and disease surveillance. It aims to improve laboratory capacity to better detect and respond to outbreaks of diseases such as Ebola virus fever, Marburg fever, tuberculosis and eruptive diseases such as mpox [8].

Several types of surveillance are being organized in the Kokolo health zone: passive surveillance, population-based surveillance, and community-based surveillance. An active search for mpox cases has been added. Simultaneous bottom-up surveillance is being conducted in health care facilities (HCFs) and in communities (churches, schools, workplaces, etc.). Every Monday, not later than 12 noon, the ESSs must report the weekly surveillance data to the Central Health Zone Office (BCZS, Bureau central de la zone de santé) using a tablet and a weekly report form. However, the reporting of any suspected case of mpox is done immediately by phone call, SMS or WhatsApp message. The Kokolo BCZS compiles all the data from the health areas and sends it to the provincial health department in Kinshasa by noon on Tuesdays. The community or ESS alert goes through the registered nurse, who investigates at his or her level and then raises the alert to the BCZS. The BCZS validates the alert or not. If the alert is invalidated, the person is treated for something other than mpox. If the alert is validated, the suspected case is isolated at an mpox treatment center and a blood, scab, or nasopharyngeal swab is taken, depending on the stage of the disease. This sample is sent to the National Institute for Biomedical Research (INRB, Institut national des recherches biomédicales) laboratory for PCR analysis. The suspect leaves the mpox treatment center after recovery or if the laboratory result is negative. Analyses are always performed at the INRB laboratory on samples collected by laboratory technicians. Results are available within a maximum of three days. This short period of time allows the implementation of public health measures: expansion of contact lists, monitoring of contacts for 21 days from the day of the last contact with the confirmed case, decontamination of places visited by the patient during the symptomatic phase, education of contacts and people close to the confirmed case, psychosocial support for contacts and people close to the confirmed case, vaccination of all contacts [2].

The present analysis is a descriptive study of data reported on the mpox epidemic in the Kokolo health zone from August 1, 2024, to November 10, 2024. They are extracted from the district's monkeypox response databases.

- Case alert: Anyone with a sudden onset of high fever that does not resolve after initial treatment, or anyone with skin or mucous membrane rashes.
- Suspected case: any person who has been exposed to a case of mpox and who has acute fever (≥38°C) or who has acute fever and symptoms of mpox, or any person who has one or more maculopapular rashes or scabs.
- Probable case: a suspected case (deceased or missing) for whom a laboratory result for mpox could not be obtained, but who has had contact cases. This is a patient for whom clinical and/or epidemiologic evidence indicates a very high probability of infection with the mpox virus, but for whom laboratory confirmation has not been obtained.
- Confirmed case: any suspected case for which a laboratory test (PCR) is positive.
- Non-case: any case for which the laboratory test is negative [2, 6].

The sample studied consists of cases registered in the response database of the Kokolo health zone. Data collection was based on the list of mpox cases in Kokolo health district and the reporting forms for diseases with epidemic potential.

This study was exempt from ethical approval because it was conducted as part of national surveillance activities and was conducted in the public interest by the Ministry of Health of the DRC. All the activities described were carried out as part of regular surveillance carried out and approved directly by the Ministry of Health. Written informed consent was not obtained because the analyses performed in this study were retrospective, using information and samples collected for surveillance and care purposes.

## Results

During the study period, 47 samples and tests were performed in August, 58 in September, 62 in October, and 35 in November 2024, for a total of 202 PCR tests for MPXV. Of the 202 tests, 123 were performed on military personnel, of whom 41 (33%) were confirmed to have mpox, 65 on their dependents, of whom 9 (14%) were confirmed to have mpox, and 14 on people living near the military camps, of whom 1 was confirmed to have mpox.

All confirmed cases of mpox presented with rashes. Other reported symptoms were myalgia (50/51), genital rash (42/51), arthralgia (42/51), and headache (41/51) (Tables I, II, III and IV). Two of the male patients admitted to mpox treatment center were HIV-positive, two cases of hypertension were identified, one case of asthma, and one case of diabetes mellitus.

**Table I:** Distribution of mpox test results by sex in the Kokolo health zone, August to November 2024

	Positive	Negative	Undetermined
**Sexe Male**	36	104	3
**Sexe Female**	15	42	2
**Total**	51 (25,2 %)	146 (72,3 %)	5 (2,5 %)

**Table II:** Distribution of people tested for mpox by age in the Kokolo health zone, August to November 2024

	Median age (years) Positive mpox result	Median age (years) Negative mpox result
**Sexe Male**	29 (IIQ : 11-31)	28 (IIQ : 17-44)
**Sexe Female**	20 (IIQ : 14-30)	19 (IIQ : 11-30)
**Total**	25 (IIQ : 11-31)	24 (IIQ : 16-44)

**Table III:** Distribution of people with confirmed mpox by age and sex in the Kokolo health zone, August to November 2024

	Sexe Male n (%)	Sexe Female n (%)	Total n (%)
**lt; 15 years**	4 (8 %)	2(4 %)	6 (12 %)
**15-30 years**	15 (29 %)	13 (25 %)	28 (54 %)
**≥30 years**	16 (31 %)	1 (2 %)	17 (34 %)

**Table IV:** : Hospitalizations, demographic data and clinical symptoms identified in the Kokolo health zone, from August to November 2024

Age groupe (years) n = 202	Sexe Male n (%)	Sexe Female n (%)	Total n (%)
**< 15 years**	17 (8,4 %)	7(3,5 %)	24 (11,9 %)
**15-30 years**	61 (30,2 %)	49 (24,3 %)	110 (4,4 %)
**≥30 years**	65 (32,2 %)	3 (1,5 %)	68 (33,7 %)
**Total**	143 (70,8 %)	59 (29,2 %)	202 (100 %)
**Median age (years)**	25 (IIQ : 4-38)	24 (IIQ : 5-35)	24 (IIQ : 4-38)
**Duration of treatment (days)**	9 (IIQ : 6-12)	11 (IIQ : 8-13)	10 (IIQ : 6-11)
**Hospitalization**	32/35	14/16	46/51 (90 %)
**Clinical symptoms n=51**			
**Skin rashes**	35	16	51 (100 %)
**Genital rashes**	29	13	42 (82 %)
**Oral rashes**	16	15	31 (61 %)
**Fever**	24	12	36 (71 %)
**Headaches**	29	12	41 (80 %)
**Myalgies**	35	15	50 (98 %)
**Arthralgies**	29	13	42 (82 %)

## Discussion

Since January 2023, the significant evolution of clade I MPXV in DRC has caused a major epidemic of MPXV in all provinces of the country. An unprecedented subclade of the virus, subclade Ib, associated with human-to-human transmission, has emerged [14]. It was first reported in the province of South Kivu. It quickly spread to North Kivu [5, 7] and to the province of Kinshasa, mainly in the Kokolo health zone, where this is the first mpox epidemic. In 2023, this health zone had recorded only two cases of mpox, including one death. The only mode of transmission recorded was human-to-human with subclade Ia. However, during the August-November 2024 episode, 51 PCR-confirmed cases belonging to the two sub-clades Ia and Ib with outbreaks in the external genital tract were recorded.

Surveillance systems are theoretically the same in all health zones of the DRC, but analysis of surveillance and response data is not always regularly updated. Previously, mpox was considered to be a disease mainly affecting children [3, 6, 13]. In our study, adults are more affected. Members of the armed forces seem to be more affected, probably because of their profession, which exposes them to all kinds of people from all places, including sex workers. The duration of hospitalization was probably shortened due to the insufficient number of mpox treatment centers in the city of Kinshasa (only 3 for a population of over 10 million people). The strengths of the monitoring system are:

- The system exists and is functional, from the community to the General Secretariat and even to the Office of the Minister of Health, with feedback at each level;
- It involves actors trained in several diseases;
- There are monitoring structures with a defined cycle.

However, this system has some limitations:

- It requires constant updating of knowledge and skills, context-specific case definitions and good coordination for greater efficiency;
- Without community buy-in, the system is doomed to failure;
- The system requires adequate resources on a continuous basis at the level of health zone central offices and health facilities.

We make recommendations at several levels:

1. Provincial health department:
  - Improve working conditions for stakeholders at all levels;
  - Update case definitions;
  - Improve coordination of response at all levels.
2. Health zone technical and financial partners:
  - Provide the Kokolo Health Zone with at least 3 Genexpert devices to cover the three areas of the zone;
  - Develop continuous and sustainable empowerment of stakeholders at all levels;
  - Provide the means of control;
  - Improve the working conditions of the actors at all levels;
  - Improve the coordination of the response at all levels.
3. Health sector:
  - Improve the working conditions of the actors;
  - Improve awareness and communication to obtain community involvement.
4. Community: Fully involve the community in mpox response activities.

These recommendations are critical to strengthening control of mpox and improving the health services at the health district level. This will require close collaboration at all levels to ensure effective and sustainable implementation.

## Conclusion

This study showed that out of 202 tests performed on persons clinically suspected of having mpox or contacts of known cases, 25.2% were positive by PCR. Adults aged 18 years and older were most affected, accounting for 80% of confirmed cases. All confirmed cases presented with rash, with other common symptoms including myalgia, genital rash, arthralgia, and headache. The median length of stay at the mpox treatment center was 10 days. The late detection of cases indicates that there are either difficulties in contact tracing or that the case definition should be revised to allow for early detection in the community and among contacts. It is also necessary to ensure that the expansion of the contact list and the follow-up of these contacts for 21 days are effective. As males, young people and often military personnel are most affected, these categories need to be targeted.

## Funding

The authors do not declare any source of funding.

## Authors’ contributions

IHEKAMBANGU NGWAKAHA Blanche: Analysis and writing.

MASAMBA BIKOKI Winnie: design, analysis and writing.

NDUMBI TEMUANGUDI Vally: design, analysis and writing.

NSINGA BUNGIENA Jean Claude: analysis and writing.

KAPE KALUME Jean-Jacques: writing.

MBUYI MUTOMBE Anthony: analysis and writing.

## Conflicts of interest

The authors declare that they have no competing interests.
